# Whole-brain input organization of *Kremen1*-positive striatal spiny projection neurons revealed by monosynaptic rabies tracing

**DOI:** 10.3389/fncir.2026.1895042

**Published:** 2026-07-29

**Authors:** Victor M. Martinez Smith, Jie Dong, Ronald F. Paletzki, Lisa Chang, Lupeng Wang, Charles R. Gerfen, Huaibin Cai

**Affiliations:** 1 Transgenics Section, Laboratory of Neurogenetics, National Institute on Aging, National Institutes of Health, Bethesda, MD, United States; 2 Department of Neurology, The First Affiliated Hospital, Zhejiang University School of Medicine, Hangzhou, China; 3 Section on Neuroanatomy, National Institute of Mental Health, National Institutes of Health, Bethesda, MD, United States

**Keywords:** basal ganglia, cortical inputs, dopaminergic modulation, dorsal striatum, Kremen1^+^ neurons, monosynaptic rabies tracing, sensorimotor integration, spiny projection neurons

## Abstract

Determining the organization of inputs to subtypes of striatal spiny projection neurons (SPNs) is essential to understand how the striatum integrates information to regulate motor control and decision making. Here, we employed monosynaptic rabies tracing in *Kremen1*^2A–Cre^ knock-in mice to map inputs to *Kremen1*-positive (*Kremen1*^+^) SPNs, a subtype enriched in patch (striosome) compartments of the dorsal striatum. Starter cells were broadly distributed along the rostrocaudal axis of the dorsal striatum. Whole-brain analysis revealed that *Kremen1*^+^ SPNs receive prominent inputs from motor, somatosensory, and prefrontal cortices, as well as from multiple thalamic nuclei. Additional inputs originated from the amygdala, basal ganglia, and midbrain regions, including dopaminergic populations. Quantitative analysis indicated a strong bias toward sensorimotor-related circuits. Together, these results provide a comprehensive whole-brain input map of *Kremen1*^+^ SPNs and suggest that these neurons are positioned to integrate cortical, thalamic, and neuromodulatory signals within basal ganglia networks to regulate motor function.

## Introduction

The dorsal striatum is organized into two interdigitated compartments, the patch (striosome) and the surrounding matrix, which differ in molecular composition, connectivity, and function and together shape basal ganglia processing of motor, cognitive, and affective behaviors (Gerfen, 1984, 1992; Graybiel, 1984; Crittenden and Graybiel, 2011). Patch striatal spiny projection neurons (SPNs) are enriched in μ-opioid receptors and exhibit distinct developmental and transcriptional profiles compared with matrix neurons, suggesting specialized circuit functions (Pert et al., 1976; Gerfen et al., 1987; Fujiyama et al., 2011; Graybiel and Matsushima, 2020). Patch SPNs contain both D1 and D2 types of SPNs and have been implicated in value-based decision-making, action evaluation, and the regulation of dopaminergic signaling in motor control (Gerfen and Young, 1988; Brimblecombe and Cragg, 2017; Friedman et al., 2020; Lazaridis et al., 2024; Dong et al., 2025; Hawes et al., 2025; Okunomiya et al., 2025). However, the afferent circuit organization underlying these functions remains incompletely defined.

Early anatomical studies indicated that both cortical and thalamic inputs contribute to striatal compartmentalization (Gerfen, 1984, 1992; Kincaid and Wilson, 1996). Corticostriatal projections exhibit graded biases rather than strict segregation: limbic and associative cortical areas, particularly from deeper layers, preferentially innervate patch compartments, whereas sensorimotor regions are biased toward the matrix (Gerfen, 1984, 1989, 1992; Kincaid and Wilson, 1996). Thalamostriatal projections further differentiate these compartments, with midline and intralaminar nuclei preferentially targeting patches, while parafascicular inputs favor the matrix (Unzai et al., 2017). These observations suggest that patch SPNs preferentially integrate motivational and contextual information. However, more recent recombinant rabies-based tracing studies have challenged this view, demonstrating that patch SPNs receive substantial and convergent inputs from sensorimotor, associative, and limbic cortical regions (Smith et al., 2016).

Patch SPNs provide prominent inhibitory projections to nigral dopaminergic neurons (DANs), particularly the aldehyde dehydrogenase 1A1 (ALDH1A1)-positive subtype (Gerfen, 1984, 1992; Watabe-Uchida et al., 2012; Crittenden et al., 2016; Wu et al., 2019; Evans et al., 2020; Lazaridis et al., 2024; Dong et al., 2025; Okunomiya et al., 2025), which broadly innervate the dorsal striatum (Sgobio et al., 2017; Poulin et al., 2018; Wu et al., 2019). Despite this reciprocal connectivity, early rabies tracing studies reported minimal dopaminergic input to patch SPNs (Smith et al., 2016), likely reflecting limitations of the viral system used. This discrepancy highlights the need for more efficient and less biased tracing approaches to accurately define presynaptic inputs to patch neurons (Reardon et al., 2016; Sumser et al., 2022). In addition, prior studies largely relied on BAC-Cre mouse lines that label both canonical patch neurons and dispersed “exo-patch” populations, potentially confounding compartment-specific interpretations (Smith et al., 2016). These limitations underscore the need for improved viral tools and molecularly defined genetic access to resolve the input organization of patch-enriched SPN subtypes.

Recent single-nucleus RNA sequencing studies have revealed substantial cellular heterogeneity within the patch compartment (Saunders et al., 2018; Martin et al., 2019). Among the molecularly distinct SPN populations identified, Kremen1, a transmembrane receptor implicated in Wnt signaling (Mao et al., 2002), marks a transcriptionally distinct striatonigral SPN subtype enriched within patch compartments (Martin et al., 2019; Dong et al., 2025). Importantly, *Kremen1*^+^ neurons comprise approximately 52% of patch SPNs and 12.7% of total dorsal striatal SPNs, indicating that they represent a major component of the patch system (Dong et al., 2025). Patch neurons, including the Kremen1^+^ subset, exert strong control over nigrostriatal dopaminergic circuits (Nadel et al., 2021; Lazaridis et al., 2024; Dong et al., 2025; Okunomiya et al., 2025). Notably, dopamine receptor D1 (Drd1)-expressing *Kremen1*^+^ SPNs directly inhibit Parkinson’s disease–vulnerable ALDH1A1-positive dopaminergic neurons through GABAergic mechanisms and modulate locomotor behavior (Liu et al., 2014; Dong et al., 2025), underscoring their importance in basal ganglia function. The substantial but selective representation of Kremen1^+^ neurons within the patch compartment makes them a valuable population for investigating whether molecularly defined patch SPN subtypes exhibit specialized patterns of brain-wide connectivity.

Despite these advances, the presynaptic input architecture of *Kremen1*^+^ SPNs remains unknown. To address this gap, we performed whole-brain monosynaptic retrograde tracing using a Cre-dependent CVS-N2c rabies system (Reardon et al., 2016; Sumser et al., 2022) in *Kremen1*^2A–Cre^ knock-in mice (Dong et al., 2025), enabling comprehensive and quantitative mapping of inputs across the brain.

## Materials and methods

### Animals

All experimental procedures were approved by the Institutional Animal Care and Use Committee (IACUC) of the Intramural Research Program at the National Institute on Aging (NIA), NIH, and were conducted in accordance with institutional and NIH guidelines. The *Kremen1*^2A–Cre^ heterozygous knock-in mice have been described previously (Dong et al., 2025) and deposited in the Jackson Laboratory (Strain#: 040610). Both male and female mice at 2–4 months of age were used in all experiments. Mice were group-housed (2–5 per cage) under a 12-h light/dark cycle with ad libitum access to water and standard chow.

### Viral constructs and stereotaxic injections

Stereotaxic surgeries were performed under aseptic conditions. Mice were anesthetized with 1–2% isoflurane and placed in a stereotaxic frame (Stoelting with a mouse &neonates Adaptor). For each mouse, a total volume of 250 nL of AAV helper virus encoding Cre-dependent TVA and rabies glycoprotein (AAV9-EF1α-DIO-TVA-3 × FLAG-N2C-G; Addgene #73478) was injected unilaterally into one of three right dorsal striatal locations along the rostrocaudal axis (coordinates relative to bregma: rostral, AP +1.4 mm, ML −1.7 mm, DV −2.7 mm; medial, AP +0.6 mm, ML −2.0 mm, DV -2.4 mm; caudal, AP −0.1 mm, ML −2.2 mm, DV −2.4 mm) of Kremen12A-Cre mice. Two weeks later, 250 nL of EnvA-pseudotyped, glycoprotein-deleted rabies virus (RVDG-CVS-N2c-tdTomato-FlpO or RVDG-CVS-N2c-EGFP-FlpO (Sumser et al., 2022); 9.70 × 10^7^ TU/ml; produced by the NIEHS Viral Vector Core) was injected at the same coordinate used for the helper-virus injection.

The three injection coordinates were selected to sample *Kremen1*^+^ SPNs across rostral, medial, and caudal levels of the dorsal striatum rather than restrict the analysis to a single anteroposterior level. Because the primary goal was to generate a representative whole-brain input map for Kremen1+ SPNs across the dorsal striatum, data from these sites were combined for the main analysis. Location-specific comparisons were performed as an exploratory analysis to evaluate whether major cortical input patterns differed across injection sites.

### Tissue processing and imaging

Tissue processing and imaging were performed following established protocols (Paletzki and Gerfen, 2019). Briefly, 7 days after rabies injection, mice were deeply anesthetized and transcardially perfused with saline followed by 4% paraformaldehyde (PFA). Brains were post-fixed overnight at 4 °C and cryoprotected in 20–30% sucrose in PBS until fully infiltrated. Coronal sections (40–50 μm) were collected across the entire rostrocaudal extent of the brain using a sliding microtome or cryostat.

Free-floating sections were processed for fluorescence imaging. Starter cells were identified as cells co-labeled by native rabies reporter fluorescence and FLAG immunostaining from the helper virus (rabbit anti-FLAG; Sigma-Aldrich, F7425; 1:1000). To avoid overlap between the rabies reporter and FLAG signal, FLAG immunostaining was detected using Alexa Fluor–conjugated secondary antibodies (Invitrogen/Thermo Fisher Scientific) selected to avoid spectral overlap with the native rabies reporter fluorescence. In experiments using RVDG-CVS-N2c-tdTomato-FlpO, FLAG was detected in a non-red channel; in experiments using RVDG-CVS-N2c-EGFP-FlpO, FLAG was detected in a non-green channel. Thus, rabies reporter fluorescence and FLAG immunostaining were always acquired in separate, non-overlapping channels. The presynaptic input neurons were identified by native fluorescence (tdTomato^+^ or EGFP^+^). To delineate patch compartments, sections were co-immunostained for (MOR1; ImmunoStar, 1:3000). For cellular identification and anatomical reference, sections were counterstained with either a fluorescent Nissl stain (NeuroTrace, Invitrogen; 1:100) or DAPI (4’,6-diamidino-2-phenylindole) prior to mounting. Images were acquired using a slide-scanning fluorescence microscope (Zeiss Axio Scan.Z1, Carl Zeiss).

### Quantification

Brain section images were processed and analyzed using *NeuroInfo* (MBF Bioscience, Williston, VT). First, serial coronal sections were imported into the software and arranged in the correct anatomical order using the Serial Section Assembler workflow. Sections were oriented with reference to the injection site to ensure proper alignment. Automated cell detection and quantification were then performed using the Detect Cells workflow. Detection parameters were set to a large diameter of 25 μm and a small diameter of 8 μm. Cell identification employed the built-in neural network–based classifier to optimize accuracy.

Experimental brain sections and the three-dimensional brain volume were registered to a reference brain atlas using NeuroInfo. For two-dimensional section registration, the Register Sections function was used. Interactive registration was first performed on a set of reference sections, defined as experimental sections containing prominent anatomical landmarks that facilitate accurate alignment to the atlas. These reference sections served as initialization points for subsequent serial registration of adjacent sections. Reference sections were selected at three anatomical locations: a rostral section where the corpus callosum first crosses the midline; a central section where the anterior commissure appears as three distinct fiber bundles; and a caudal section corresponding to the first appearance of a rounded fasciculus retroflexus. Following verification of accurate alignment, the remaining sections were serially registered.

The three-dimensional brain volume was then registered using the Register Volume function. A location near the center of the brain volume was used as a reference. Registration was performed using both linear and nonlinear transformations. Linear registration was performed first to establish global alignment between the experimental image and the atlas, followed by nonlinear registration to improve local alignment. Registration accuracy was evaluated throughout the brain volume. Final registration quality was assessed by visual inspection of anatomical alignment across the entire dataset before downstream analyses were performed. Representative examples of Allen Brain Atlas boundaries overlaid onto experimental sections are shown in Supplementary Figure 3. More comprehensive details regarding the NeuroInfo registration workflow are available on the NeuroInfo website.

After registration, the output data consisted of the coordinates for each detected cell and a summary table reporting the number of cells within each brain region as defined by the Allen Brain Atlas. To enable comparisons across animals, input counts were normalized to the total number of presynaptic input neurons detected in each brain and are reported as the percentage of total inputs per region. This normalization minimizes variability arising from differences in starter cell number and labeling efficiency across animals.

To generate a three-dimensional (3D) video of a registered volume in NeuroInfo, the 3D visualization window was first accessed via the “Registration,” “Image,” or “Workspace” ribbon located at the top of the user interface. Within the 3D visualization window, video clips were created using the Movie Mode workflow. This workflow enabled the specification of temporal positions and viewpoints for the volume, which were subsequently compiled and exported as a video file.

### Statistics

All analyses were performed using data from eight mice unless otherwise stated. Anatomically registered datasets were visualized using BrainGlobe/brainrender (GitHub) (Claudi et al., 2021); statistical analyses were performed separately using GraphPad Prism 10 (GraphPad Software). Data are reported as mean +/− standard error of the mean (SEM) unless otherwise indicated. For comparisons between two groups, unpaired two-tailed *t*-tests were used. For comparisons among three or more groups, one-way ANOVA followed by post hoc multiple-comparison tests was used. The exact statistical test used for each analysis, including degrees of freedom, test statistics, sample size, and *p* values, is provided in the corresponding figure legends.

## Results

### Kremen1^+^ starter cells

**FIGURE 1:**
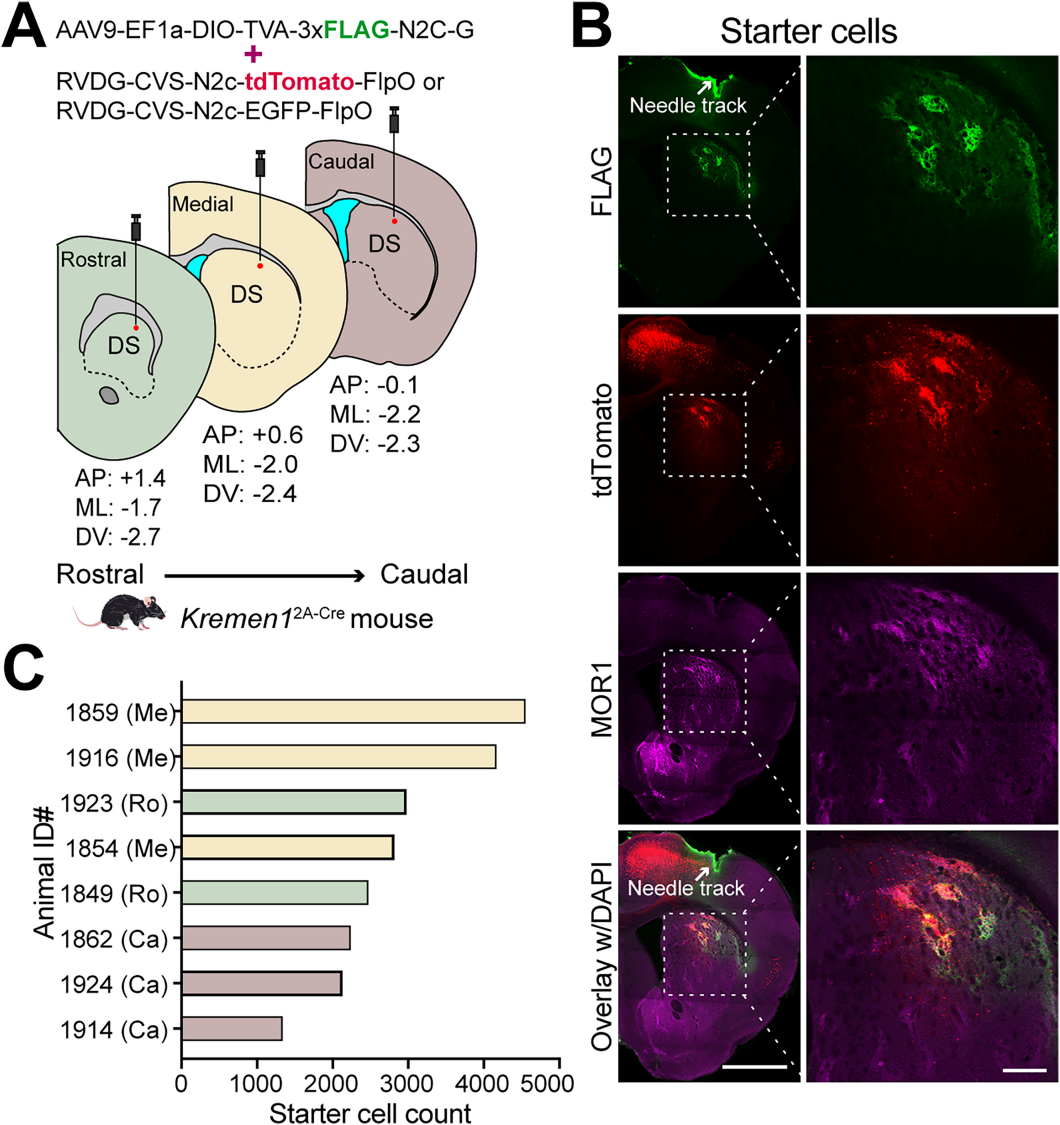
Distribution of *Kremen1*^+^ starter cells in the dorsal striatum. **(A)** Schematic of experimental design showing unilateral injection of Cre-dependent helper AAVs and EnvA-pseudotyped CVS-N2c rabies virus into one of three rostrocaudal locations of the dorsal striatum in each *Kremen1*^2A–Cre^ mouse. **(B)** Representative coronal sections showing *Kremen1*^+^ starter cells within patch compartments. Starter cells were identified by co-localization of native rabies reporter fluorescence, either tdTomato or EGFP, and FLAG immunostaining from the helper virus, acquired in spectrally distinct channels. MOR1 immunostaining was used to identify patch compartments. Separated channels and merged images are shown. Scale bars: 1000 μm (left), 200 μm (right). **(C)** Quantification of starter cell distribution across rostral (Ro), medial (Me), and caudal (Ca) levels of the dorsal striatum.

Helper AAVs and pseudotyped recombinant rabies virus were unilaterally injected into one of three sites along the rostrocaudal axis of the dorsal striatum in each *Kremen1*^2A–Cre^ mouse (Figure 1A). The *Kremen1*^+^ starter cells, defined by co-expression of FLAG and fluorescent reporters (EGFP or tdTomato) from the helper virus and rabies vector, were largely confined to patch compartments, as delineated by MOR1 immunostaining (Figure 1B). Consistent with selective transgene expression in patch compartments in *Kremen1*^2A–Cre^ mice, in some cases the brighter rabies-derived fluorescence was used to identify starter cells in the dorsal striatum.

Across animals, the number of starter cells ranged from approximately 1,300 to 4,600 per brain and were distributed across rostral (*n* = 2), middle (*n* = 3), and caudal (*n* = 3) levels of the dorsal striatum (Figure 1C). Whole-brain image datasets from all eight mice are publicly available through the Biolucida platform (https://gerfenc.biolucida.net/image_view/#;dataset:jHC-Huaibin Cai). A higher density of starter cells was observed in medial regions compared to rostral and caudal regions, consistent with the larger volume of the medial dorsal striatum (Figure 1C). Notably, starter cells frequently formed clusters, which in some cases complicated accurate segmentation and counting; therefore, the reported numbers likely underestimate the true number of starter cells in some animals.

Occasional FLAG immunoreactivity was observed in the cortex along the injection needle track. Higher-magnification examination indicated that this signal was mainly associated with tissue surrounding the needle track and did not show clear neuronal morphology (Supplementary Figure 2A). In addition, previous characterization of *Kremen1*^2A–Cre^; Ai14 reporter mice showed that Kremen1-positive cells are predominantly distributed in the dorsal striatum and hippocampus, with sparse cortical labeling, if any (Supplementary Figure 2B; Dong et al., 2025). We did not observe cortical cells co-labeled with FLAG and rabies reporter fluorescence, suggesting that cortical starter cells, if present, were negligible.

### Whole-brain input distribution

**FIGURE 2:**
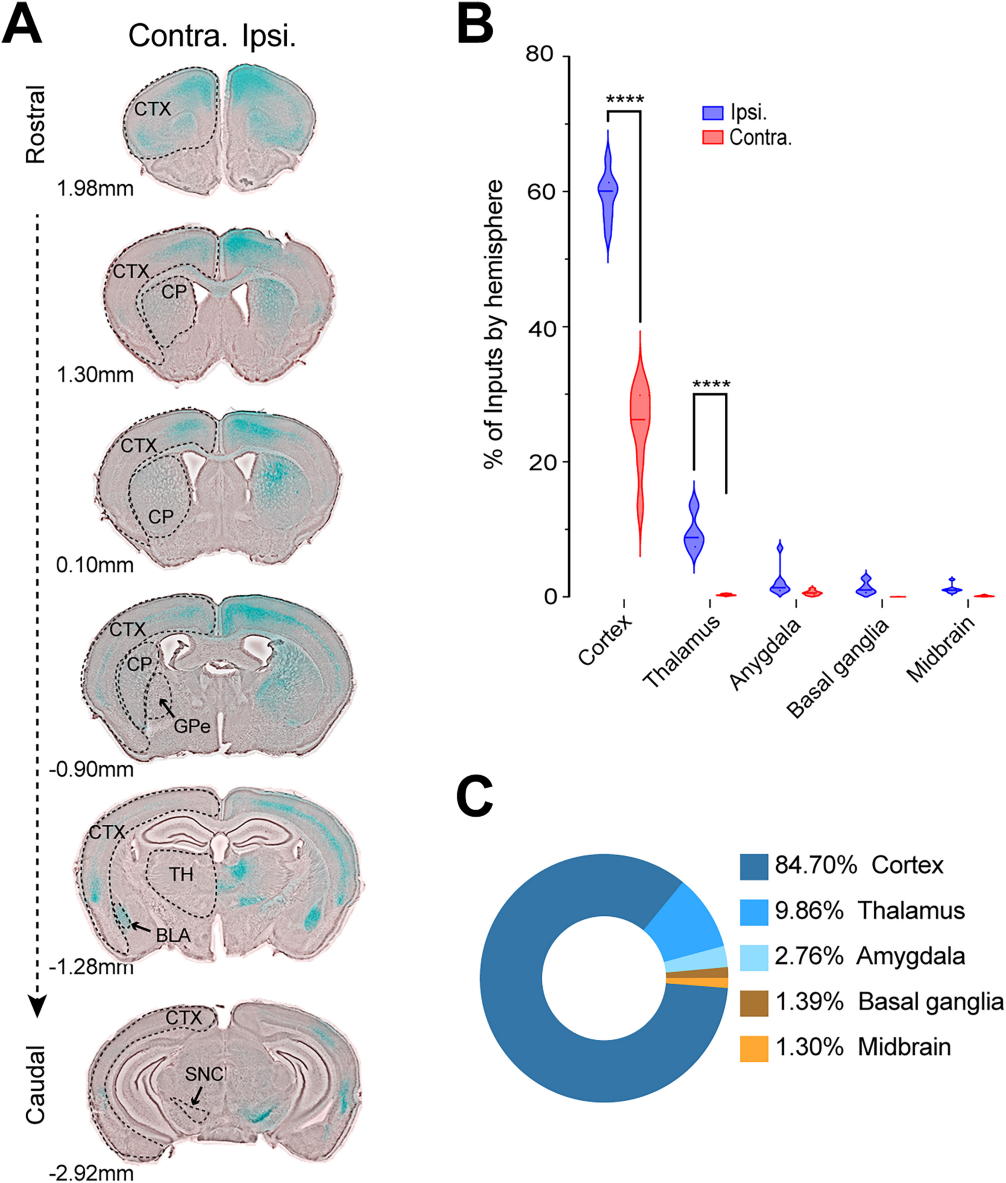
Whole-brain distribution of inputs to *Kremen1*^+^ SPNs. **(A)** Representative coronal sections, annotated with estimated Bregma coordinates, illustrating rabies-labeled presynaptic neurons (tdTomato^+^) across the outlined brain regions. CTX: cerebral cortex; CP: caudoputamen; GPe: globus pallidus, external segment; TH: thalamus; BLA: basolateral amygdala; SNc: substantia nigra pars compacta. Scale bar: 1000 μm. **(B)** Quantification of ipsilateral versus contralateral input distribution, showing a strong ipsilateral bias. Violin plot shows median and quantile. *N* = 8 mice. Two-way ANOVA, multiple comparisons: cortex, ****p < 0.0001; thalamus, ****p = < 0.0001; amygdala, *p* = 0.7147; basal ganglia, *p* = 0.7987; midbrain, *p* = 0.9298. **(C)** Brain-wide distribution of bilateral inputs by major regions. Data are normalized to the total number of input neurons per brain and expressed as percentage contribution. The cortex provides most inputs, followed by thalamus, amygdala, basal ganglia, and midbrain. *N* = 8 mice.

Rabies-labeled presynaptic input neurons, identified by EGFP or tdTomato expression, were detected across serial coronal sections spanning the entire mouse brain (Figure 2A, Supplementary Video). The dorsal striatum was excluded from input quantification due to the difficulty in reliably distinguishing starter from input cells. The spatial distribution and number of input neurons were reconstructed and quantified using NeuroInfo and subsequently registered to the Allen Brain Atlas for classification into anatomical regions and subregions (Supplementary Figure 3). Overall, Kremen1^+^ patch SPNs received inputs from both hemispheres, with a strong predominance from the ipsilateral hemisphere (Figure 2B). Quantitative analysis, normalized to the total number of input neurons per brain, revealed that the cerebral cortex contributed the majority of inputs (∼85%), followed by the thalamus (∼10%), amygdala (∼3%), basal ganglia (∼1%), and midbrain (∼1%) (Figure 2C). Together, these findings indicate that Kremen1^+^ patch SPNs are embedded within cortex-dominated input networks, with additional contributions from thalamic, limbic, and other subcortical regions.

### Cortical inputs

**FIGURE 3:**
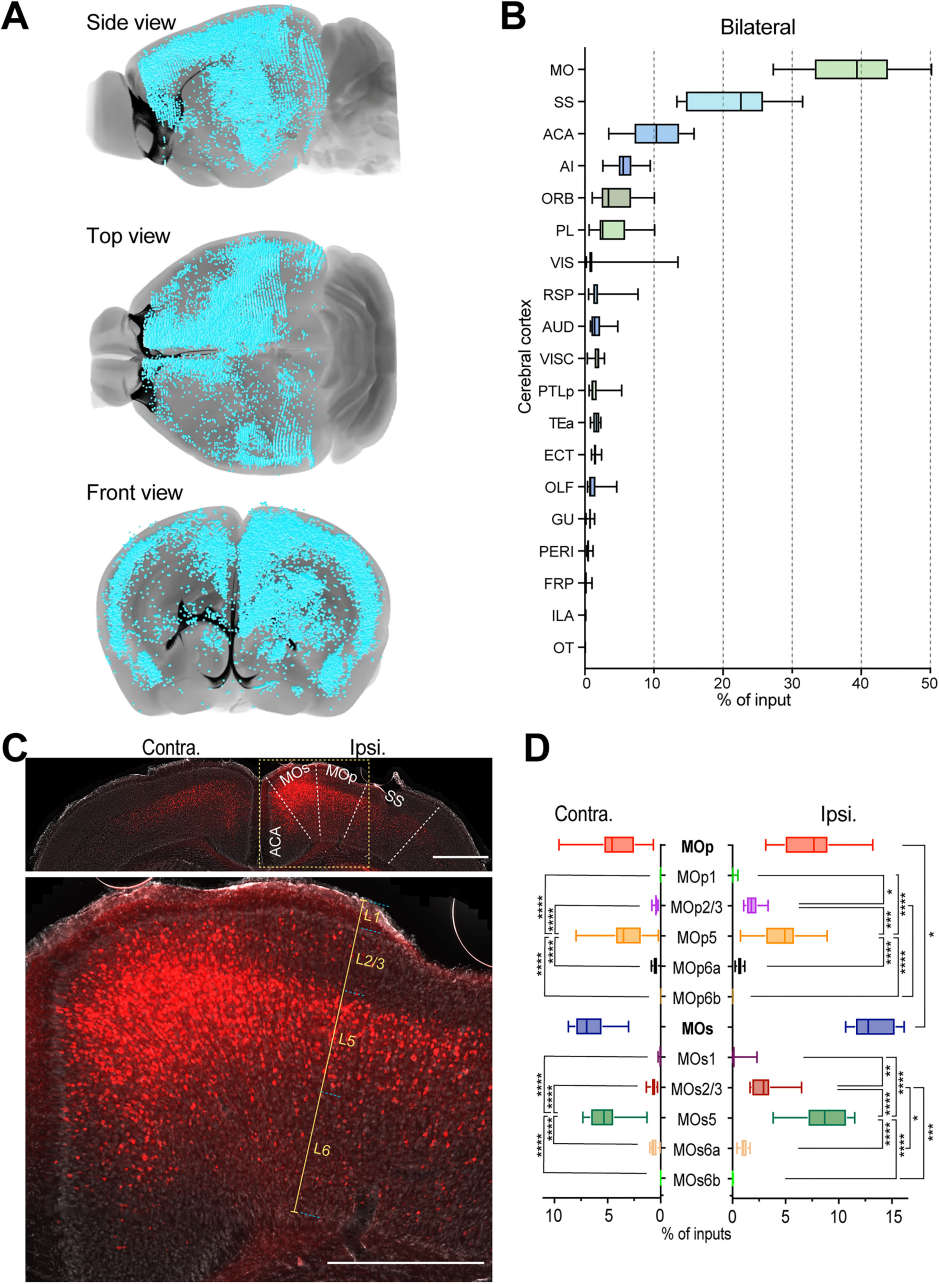
Cortical inputs to *Kremen1*^+^ SPNs. **(A)** Snapshots from the whole-brain reconstruction (see Supplementary Video) showing the distribution of cortical inputs (blue dots) to *Kremen1*^+^ SPNs, compiled across all animals and visualized from multiple viewing angles. **(B)** Quantification of the percentage of total cortical inputs contributed by each region, noting that cortical inputs account for ∼80% of total brain inputs, with dominant contributions from motor (MO) and somatosensory (SS) cortices and additional input from prefrontal and associative areas (ACA, PL, ORB, AI). **(C)** Representative cortical images illustrating the laminar distribution of rabies-labeled cortical input neurons. The top panel shows a low-magnification coronal section containing both ipsilateral and contralateral hemispheres. Bottom panels show higher-magnification views of motor and sensory cortical regions from the ipsilateral hemisphere. Cortical regions, including anterior cingulate area (ACA), secondary motor cortex (MOs), primary motor cortex (MOp), and somatosensory cortex (SS), are labeled. Cortical layer boundaries for layers 1, 2/3, 5, and 6 are delineated based on anatomical landmarks and Allen Brain Atlas registration. Scale bars: 1000 μm, low-magnification image; 500 μm, higher-magnification images. **(D)** Laminar and projection-class analysis of ipsilateral and contralateral inputs, where ipsilateral inputs from the secondary motor areas are dominant, unpaired *t*-test, two-tailed, *p = 0.0759; *n* = 8 mice. Inputs are enriched in layer 5 across hemispheres neurons and predominantly arise from intratelencephalic (IT) and pyramidal tract (PT) projection neurons in layer 5 and layers 2/3, with minimal corticothalamic (CT) contribution in layer 6. Data are shown as box plots (min to max; *n* = 8 mice). One-way ANOVA: Contralateral primary motor area: F(4, 35) = 15.68, *p* ≤ 0.0001; Contralateral secondary motor area: F(4, 35) = 49.91, *p* ≤ 0.0001; Ipsilateral primary motor area: F(4, 35) = 22.69, *p* ≤ 0.0001; Ipsilateral secondary motor area: F(4, 35) = 51.58, *p* ≤ 0.0001. Multiple comparisons: Contralateral primary motor area: layer 1 vs. layer 5, ****p ≤ 0.0001; layer 2/3 vs. layer 5 ****p ≤ 0.0001; layer 5 vs. layer 6a, ****p ≤ 0.0001; layer 5 vs. layer 6b, ****p ≤ 0.0001; Contralateral secondary motor area: layer 1 vs. layer 5****p ≤ 0.0001; layers 2/3 vs. layer 5, ****p ≤ 0.0001, layer 5 vs. layer 6a, ****p ≤ 0.0001; layer 5 vs. layer 6b, ****p ≤ 0.0001. Ipsilateral primary motor area: layer 1 vs. layer 2/3, *p ≤ 0.0245; layer 1 vs. layer 5, ****p ≤ 0.0001; layer 2/3 vs. layer 5, ***p ≤ 0.0003; layer 2/3 vs. layer 6b, *p ≤ 0.0172; layer 5 vs. layer 6a, ****p ≤ 0.0001; layer 5 vs. layer 6b, ****p ≤ 0.0001. Ipsilateral secondary motor area: layer 1 vs. layers 2/3, **p ≤ 0.0039; layer 1 vs. layer 5, ****p ≤ 0.0001; layers 2/3 vs. layer 5, ****p ≤ 0.0001; layers 2/3 vs. layer 6a, *p = 0.0441, layers 2/3 vs. 6b, ***p = 0.0008; layer 5 vs. layer 6a, ****p ≤ 0.0001; layer 5 vs. layer 6b, ****p ≤ 0.0001.

We analyzed the distribution of presynaptic inputs across cortical regions (Figure 3A). Rabies-labeled neurons were broadly distributed throughout the cortex, indicating extensive corticostriatal connectivity. Quantitative analysis revealed that motor (MO) and somatosensory (SS) cortices constituted the dominant sources of input within the striatum (Figure 3B). In addition to these sensorimotor regions, substantial inputs were observed from prefrontal and other associative cortices, including the anterior cingulate area (ACA), prelimbic area (PL), orbital cortex (ORB), and agranular insular area (AI), whereas visual (VIS) and auditory (AUD) cortices contributed relatively minor inputs (Figure 3B).

Within the motor cortex, inputs were strongly enriched in the secondary motor area (MOs), which contributed substantially more than the primary motor area (MOp) (Figures 3C, D). To illustrate the laminar distribution of cortical inputs, representative images show both hemispheres and higher-magnification views with cortical areas and layer boundaries delineated according to anatomical landmarks and Allen Brain Atlas registration (Figure 3C). Although labeled neurons were visible in layers 2/3, particularly in MOs, quantitative analysis across animals showed that cortical inputs predominantly originated from layer 5 neurons, with smaller contributions from layers 2/3 and minimal input from layer 6 (Figure 3D). Layer 5 inputs are consistent with contributions from corticostriatal projection neurons, including bilateral intratelencephalic (IT) projections and ipsilateral pyramidal tract (PT) projections. Thus, contralateral cortical inputs most likely arise from IT neurons, whereas PT-derived inputs are restricted to the ipsilateral hemisphere.

Together, these results indicate that *Kremen1*^+^ patch SPNs preferentially receive inputs from higher-order motor cortical regions, primarily originating from layer 5 IT and PT projection neurons, supporting a role in integrating motor and associative cortical signals within basal ganglia networks.

To address whether pooling across the three injection sites might mask subregion-specific differences in cortical input organization, we separately quantified the top five bilateral cortical input regions for rostral (*n* = 2), medial (*n* = 3), and caudal (*n* = 3) injection sites (Supplementary Figure 1). Across these injection locations, the relative contributions of the major cortical input regions, including somatomotor, somatosensory, anterior cingulate, agranular insular, and orbital areas, showed no significant site-dependent differences. Therefore, we combined data across the three injection sites for the whole-brain analyses while interpreting the convergent cortical input pattern with appropriate caution. Because the number of animals per injection location was limited, this exploratory analysis was not powered to detect subtle rostrocaudal differences; future studies with larger cohorts targeted to each striatal subregion will be required to define fine-scale anatomical differences among *Kremen1*^+^ SPNs in distinct striatal subregions.

### Thalamic inputs

**FIGURE 4:**
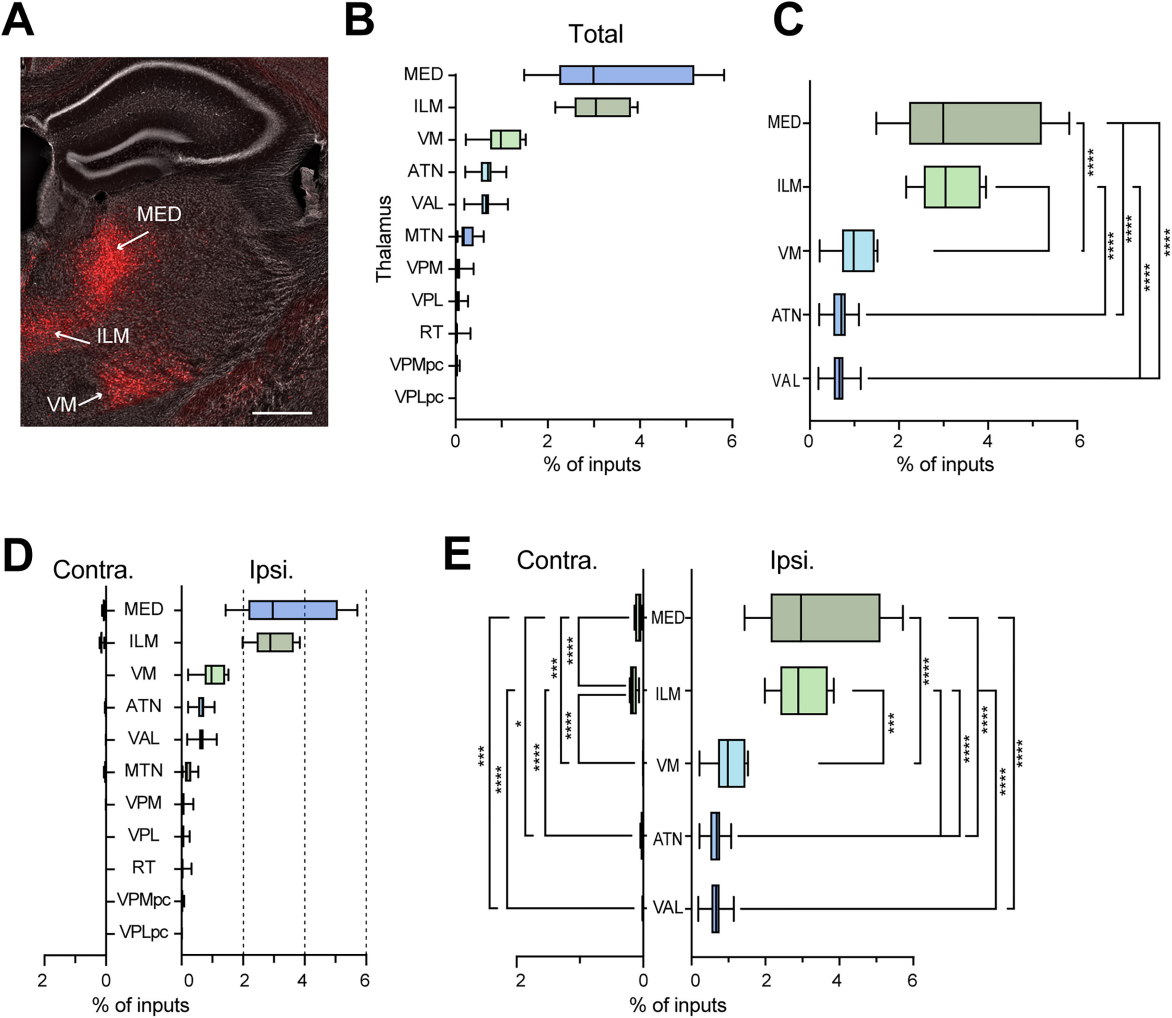
Thalamic inputs to *Kremen1*^+^ SPNs. **(A)** Representative image showing the distribution of rabies-labeled neurons (tdTomato^+^) across thalamic nuclei. Scale bar: 1000μm. **(B)** Quantification of inputs from individual thalamic nuclei, showing dominant contributions from medial group of the dorsal thalamus (MED) and intralaminar nuclei of dorsal thalamus (ILM) nuclei, with additional inputs from ventromedial (VM), anterior group of dorsal thalamus (ATN), ventral anterior-lateral (VAL), and midline group of the dorsal thalamus (MTN) nuclei. VPM: ventral posteromedial nucleus; VPL: ventral posterolateral nucleus; RT: reticular nucleus; VPMpc: ventral posteromedial nucleus/parvicellular part; VPLpc: ventral posterolateral nucleus/parvicellular part. **(C)** Comparison of the top five ipsilateral and contralateral thalamic nuclei area inputs with a one-way ANOVA: Contralateral thalamus: F(4, 35) = 37.72, *p* ≤ 0.0001; Ipsilateral thalamus: F(4, 35) = 20.95, *p* ≤ 0.0001. Multiple comparisons: Contralateral thalamus: MED vs. ILM, ****p ≤ 0.0001; MED vs. VM, ***p = 0.0004; MED vs. ATN, *p ≤ 0.0112; MED vs. VAL, ***p = 0.0004; ILM vs. VM, ****p ≤ 0.0001; ILM vs. ATN, ****p ≤ 0.0001; ILM vs. VAL, ****p ≤ 0.0001. Ipsilateral thalamus: MED vs. VM, ****p ≤ 0.0001; MED vs. ATN, ****p ≤ 0.0001; MED vs. VAL, ****p ≤ 0.0001; ILM vs. VM, *p* ≤ 0.0002; ILM vs. ATN, ****p ≤ 0.0001; ILM vs. VAL, ****p ≤ 0.0001. **(D)** Comparison of ipsilateral and contralateral inputs, demonstrating consistent input patterns across levels. Data are shown as box plots (min to max; *n* = 8 mice). **(E)** Comparison of the top five ipsilateral and contralateral thalamic nuclei area inputs with a one-way ANOVA: Contralateral thalamus: F(4, 35) = 37.72, *p* ≤ 0.0001; Ipsilateral thalamus: F(4, 35) = 20.95, *p* ≤ 0.0001. Multiple comparisons: Contralateral thalamus: MED vs. ILM, ****p ≤ 0.0001; MED vs. VM, ***p = 0.0004; MED vs. ATN, *p ≤ 0.0112; MED vs. VAL, ***p = 0.0004; ILM vs. VM, ****p ≤ 0.0001; ILM vs. ATN, ****p ≤ 0.0001; ILM vs. VAL, ****p ≤ 0.0001. Ipsilateral thalamus: MED vs. VM, ****p ≤ 0.0001; MED vs. ATN, ****p ≤ 0.0001; MED vs. VAL, ****p ≤ 0.0001; ILM vs. VM, *p* ≤ 0.0002; ILM vs. ATN, ****p ≤ 0.0001; ILM vs. VAL, ****p ≤ 0.0001.

We next analyzed thalamic inputs to *Kremen1*^+^ patch SPNs (Figure 4A). Rabies-labeled neurons were distributed across multiple thalamic nuclei, indicating broad thalamostriatal innervation. Quantification revealed that inputs were not uniformly distributed but were dominated by specific nuclei (Figures 4B, C). Among these, the medial group of the dorsal thalamus (MED) and intralaminar nuclei of dorsal thalamus (ILM) constituted the major sources of thalamic input. Additional contributions were observed from ventral motor-related nuclei, including the ventromedial (VM) and ventral anterior-lateral (VAL) complexes, as well as from midline nuclei such as the midline thalamic nuclei (MTN). Sensory-related nuclei, including the posterior (PO) and ventral posterior (VP) groups, provided comparatively minor inputs. Across hemispheres, similar patterns of thalamic innervation were observed (Figures 4D, E).

These results indicate that *Kremen1*^+^ patch SPNs receive convergent thalamic inputs primarily from intralaminar and associative nuclei, with additional contributions from motor-related thalamic regions, suggesting integration of arousal-, associative-, and motor-related signals within striatal patch circuits.

### Amygdala inputs

**FIGURE 5:**
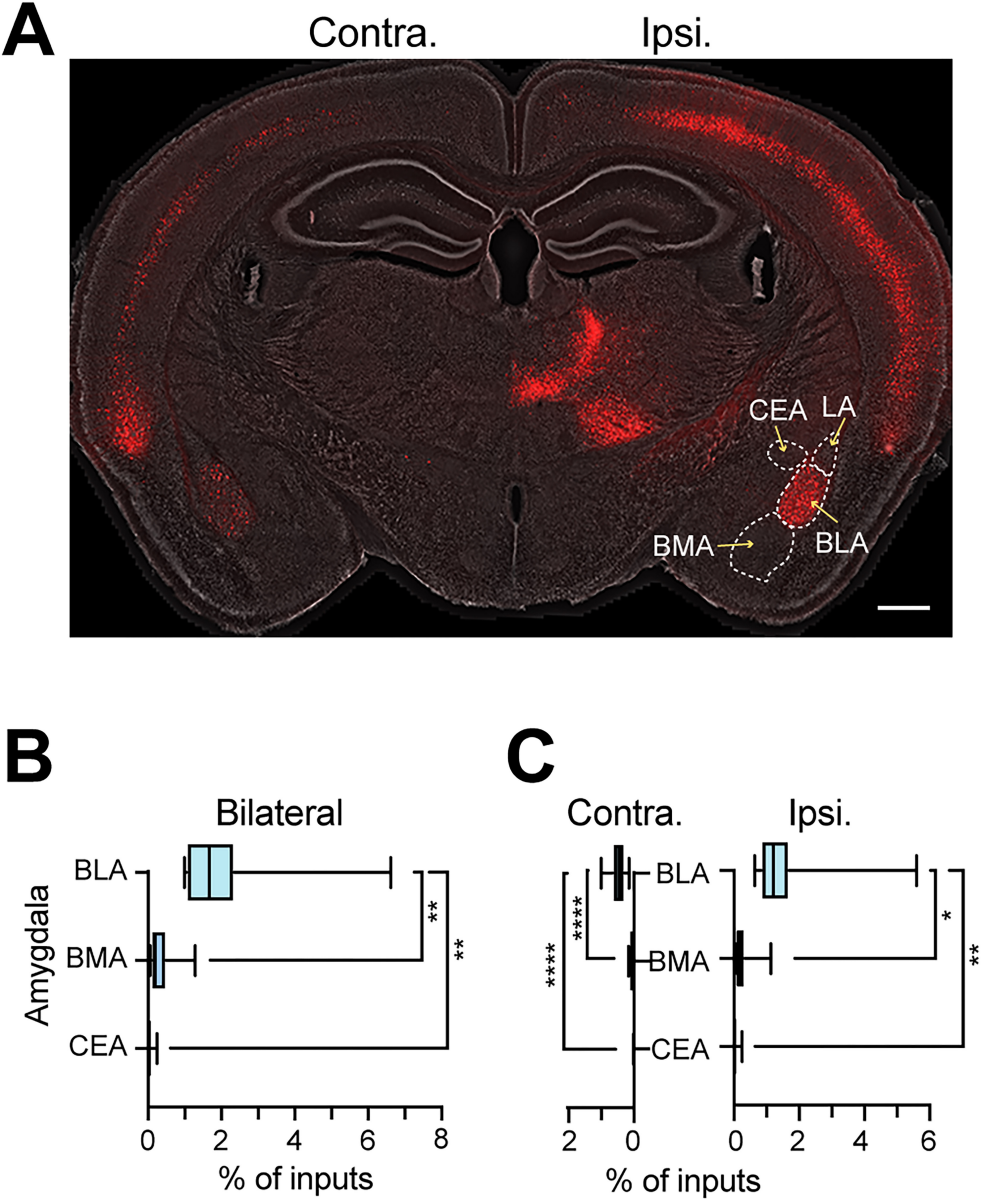
Amygdala inputs to *Kremen1*^+^ SPNs. **(A)** Representative coronal images from ipsilateral and contralateral hemispheres showing rabies-labeled presynaptic input neurons within amygdala subregions. Boundaries of the basolateral amygdala (BLA), basomedial amygdala (BMA), and central amygdala (CEA) are delineated based on anatomical landmarks and Allen Brain Atlas registration. Scale bar: 500 μm. **(B)** Bilateral quantification of amygdala inputs, showing enrichment in BLA, followed by BMA and CEA. One-way ANOVA: F(2, 21) = 9.093, *p* = 0.0014. Multiple comparisons: BLA vs. BMA, **p = 0.0073; BLA vs. CEA, **p = 0.0021. **(C)** Comparison of ipsilateral and contralateral inputs in amygdala. Data are shown as box plots (min to max; *n* = 8 mice). One-way ANOVA: Contralateral amygdala: F(2, 21) = 23.38, *p* ≤ 0.0001; Ipsilateral amygdala: F(2, 21) = 7.043, *p* = 0.0046. Multiple comparisons: Contralateral amygdala: BLA vs. BMA, ****p ≤ 0.0001; BLA vs. CEA, ****p ≤ 0.0001. Ipsilateral amygdala: BLA vs. BMA, *p = 0.0180; BLA vs. CEA, **p = 0.0064.

We next examined inputs from the amygdala to *Kremen1*^+^ patch SPNs (Figure 5A). Rabies-labeled neurons were detected in multiple amygdala subregions, indicating that these neurons receive limbic inputs. Bilateral quantification revealed that inputs were strongly enriched in the basolateral amygdala (BLA), which constituted the predominant source of amygdala input (Figure 5B). Because the rabies injections were unilateral, we further analyzed ipsilateral and contralateral amygdala inputs separately to assess input laterality. This analysis showed that the BLA remained the dominant amygdala input source across hemispheres, although overall contralateral labeling was lower (Figure 5C). Thus, the bilateral and hemisphere-specific analyses provide complementary information: the former defines the major amygdala input sources, whereas the latter evaluates the laterality and consistency of these inputs.

### Basal ganglia and midbrain inputs

**FIGURE 6:**
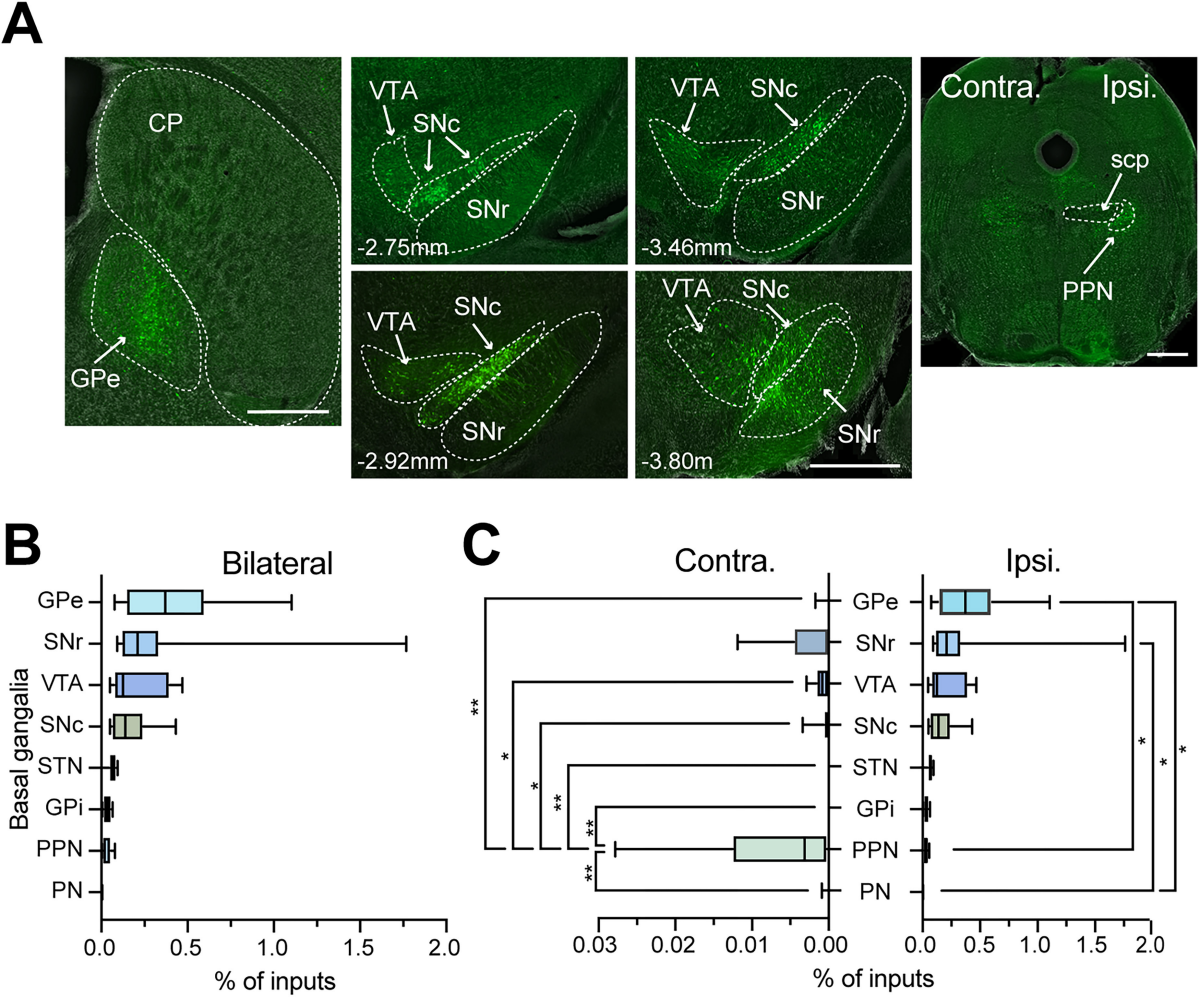
Basal ganglia and midbrain inputs to *Kremen1*^+^ SPNs. **(A)** Representative images showing the distribution of rabies-labeled neurons (EGFP) across basal ganglia and midbrain regions, including the caudoputamen (CP), external globus pallidus (GPe), substantia nigra pars reticulata (SNr), substantia nigra pars compacta (SNc), ventral tegmental area (VTA), and pedunculopontine nucleus (PPN). The superior cerebellar peduncle (scp) is outlined as an anatomical landmark for identifying the PPN, which is located lateral to the scp. The midbrain sections are annotated with estimated Bregma coordinates. Scale bars: 500 μm. **(B)** Quantification of inputs, showing the largest contribution from the GPe, followed by SNr, SNc, VTA, and smaller contributions from subthalamic nucleus (STN). GPi: internal globus pallidus; PN: paranigral nucleus. One-way ANOVA: F(7, 56) = 3.584, *p* = 0.0030. Multiple comparisons: PN vs. SNr, *p = 0.0413; PN vs. GPe, *p = 0.0262. **(C)** Comparison of ipsilateral and contralateral inputs, showing reduced contralateral labeling with relatively greater contributions from SNr and PPN. Data are shown as box plots (min to max; *n* = 8 mice). One-way ANOVA: Contralateral basal ganglia: F(7, 56) = 3.507, *p* = 0.0035; Ipsilateral basal ganglia: F(7, 56) = 3.601, *p* = 0.0029. Multiple comparisons: PN vs. PPN, **p = 0.0084; PPN vs. GPi, **p = 0.0070; PPN vs. STN, **p = 0.0070; PPN vs. SNc, *p = 0.0162; PPN vs. VTA, *p = 0.0302; PPN vs. GPe, **p ≤ 0.0100. Ipsilateral basal ganglia: PPN vs. GPe, *p = 0.0492; PN vs. SNr, *p = 0.0432; PN vs. GPe, *p = 0.0260.

We finally examined inputs from basal ganglia and associated midbrain nuclei to *Kremen1*^+^ patch SPNs (Figure 6A). Although these inputs comprised a relatively small fraction of the total input population, they exhibited a distinct and consistent distribution across nuclei.

Quantitative analysis revealed that the largest contribution arose from the external segment of the globus pallidus (GPe), followed by inputs from the midbrain areas with dopaminergic neurons including the substantia nigra pars reticulata (SNr), substantia nigra pars compacta (SNc), and ventral tegmental area (VTA), with comparatively smaller inputs from the subthalamic nucleus (STN) and other nuclei (Figure 6B).

Comparison across hemispheres showed that contralateral inputs were substantially reduced overall, with relatively greater contributions from SNr and the pedunculopontine nucleus (PPN) (Figure 6C).

These findings indicate that *Kremen1*^+^ patch SPNs receive preferential inputs from pallidal and nigral circuits, along with contributions from dopaminergic midbrain nuclei, suggesting integration of intra-basal ganglia and neuromodulatory signals within patch-specific striatal networks. Together, these results demonstrate that *Kremen1*^+^ patch SPNs integrate distributed cortical, thalamic, limbic, and subcortical inputs, forming a convergent network that spans multiple functional domains.

## Discussion

In this study, we provide a whole-brain map of presynaptic inputs to *Kremen1*^+^ SPNs, a molecularly defined patch-enriched neuron population. The major input sources identified here, including cortical, thalamic, amygdala, basal ganglia, and midbrain regions, are broadly consistent with established striatal connectivity literature and previous patch/matrix tracing studies. Thus, the significance of the present work lies not in identifying entirely unexpected input sources, but in defining the relative organization of these afferents onto *Kremen1*^+^ SPNs specifically. By combining genetic targeting with whole-brain monosynaptic rabies tracing, our findings refine prior models of striatal compartmental organization and provide a quantitative framework for understanding how this molecularly defined SPN subtype is embedded within brain-wide basal ganglia circuits. Accordingly, these data should be interpreted as defining the input architecture of *Kremen1*^+^ SPNs within a broader striatal framework in which many SPN populations receive convergent cortical and subcortical inputs.

*Kremen1*^+^ SPNs receive strong inputs from motor and somatosensory cortices, with additional contributions from prefrontal and other associative areas. This organization is consistent with the topographic yet overlapping nature of corticostriatal projections (Kincaid and Wilson, 1996; Smith et al., 2016). The enrichment of layer 5 inputs further indicates a bias toward corticofugal projection neurons, suggesting that these patch neurons preferentially integrate signals related to motor output and action execution. Although patch compartments have traditionally been associated with limbic inputs, whereas matrix compartments are linked to sensorimotor cortex (Gerfen, 1984, 1992), our data support a more integrative model in which patch neurons process both motivational and sensorimotor information. Such convergence may enable these neurons to link internal state representations with ongoing motor programs.

Thalamic inputs represent a major subcortical component of the *Kremen1*^+^ SPN input network. Thalamostriatal projections exhibit compartment-specific synaptic organization, targeting distinct dendritic domains in patch versus matrix neurons (Raju et al., 2006), suggesting differential regulation of excitability and plasticity. The diversity of thalamic nuclei identified here indicates that *Kremen1*^+^ neurons integrate sensorimotor, associative, and state-dependent signals. Together with cortical inputs, this supports a model in which patch SPNs function as multimodal integrators within basal ganglia circuits.

Amygdala inputs provide an additional limbic component to the afferent network of *Kremen1*^+^ SPNs. We observed that these inputs arise predominantly from the BLA, with smaller contributions from basomedial and central nuclei. The BLA is a major source of associative and emotional information and plays a key role in encoding valence, reward prediction, and action–outcome associations (Janak and Tye, 2015). Its preferential input to *Kremen1*^+^ neurons suggests that patch circuits are well positioned to integrate emotional and motivational signals with ongoing motor and cognitive processes. Functionally, the convergence of amygdala, cortical, and thalamic inputs onto *Kremen1*^+^ neurons supports a model in which these cells transform affective and contextual information into modulatory control of motor output. Amygdala-derived signals may convey environmental valence or emotional salience, which can be integrated within patch circuits to shape context-dependent behavioral responses.

Inputs from basal ganglia and midbrain regions further position *Kremen1*^+^ neurons within recurrent cortico–basal ganglia–midbrain loops. Patch SPNs preferentially project to ALDH1A1^+^ DANs in the SNc, forming a key feedback pathway that regulates dopamine release (Gerfen, 1985; Crittenden et al., 2016; Wu et al., 2019). Recent work demonstrates that *Kremen1*^+^ striatonigral neurons suppress locomotion by inhibiting ALDH1A1^+^ dopaminergic neurons via GABA-B receptor–dependent mechanisms (Dong et al., 2025). Our results extend this framework by showing that *Kremen1*^+^ neurons receive broad cortical and thalamic inputs, a feature shared by many SPN populations, and by placing these expected afferents in the context of a patch-enriched subtype with known projections to ALDH1A1^+^ DANs. Thus, the functional relevance of this connectivity lies in the combination of broad striatal afferent input with the molecular identity and dopaminergic output relationships of *Kremen1*^+^ SPNs, rather than in broad integration per se.

Importantly, our findings align with recent functional studies demonstrating that patch striatonigral neurons regulate locomotor vigor rather than simply movement initiation. In particular, these neurons constrain movement speed in response to environmental valence, encode deceleration, and modulate behavior during state transitions (Hawes et al., 2025). When considered together with prior functional data, this input map suggests that *Kremen1*^+^ neurons may contribute to the transformation of cortical and thalamic signals into graded control of motor output, while acknowledging that broad afferent convergence is a general feature of striatal SPNs. In this framework, cortical inputs provide motor commands and contextual information, thalamic inputs convey state-dependent signals, and dopaminergic inputs support learning and reinforcement. Together, these signals may be integrated to regulate how vigorously an action is executed, rather than whether it occurs. This expands the functional role of patch circuits beyond binary action selection to include continuous modulation of behavioral output.

These studies challenge the classical view that direct-pathway (striatonigral) neurons uniformly promote movement. Both Kremen1-based studies and recent functional work indicate that specific patch-associated dSPNs can suppress locomotion or terminate ongoing movement (Lazaridis et al., 2024; Dong et al., 2025; Hawes et al., 2025; Okunomiya et al., 2025). This suggests that functional output depends not only on pathway identity but also on molecular subtype and circuit context. *Kremen1*^+^ neurons likely represent a specialized striatonigral subpopulation that integrates distributed inputs and modulates both dopamine release and motor output, highlighting the importance of molecular heterogeneity in basal ganglia function.

Given their connectivity with DANs and their role in regulating movement vigor, *Kremen1*^+^ patch neurons may be particularly relevant to neurological and psychiatric disorders. In Parkinson’s disease and Huntington’s disease, dysfunction of these circuits could contribute not only to deficits in movement initiation but also to abnormalities in movement scaling, motivation, and behavioral flexibility. More broadly, the ability of patch neurons to link environmental valence to action vigor suggests potential roles in neuropsychiatric conditions involving altered motivation, such as depression and anxiety disorders.

Compared with earlier rabies-based studies, our approach differs in both viral strategy and genetic targeting. Smith et al. (2016) used an EnvA-pseudotyped SAD-B19/dG rabies virus in a BAC-Cre mouse line (GENSAT: Sepw1_NP67) to map inputs to patch and matrix compartments. Despite broad agreement that patch SPNs receive extensive cortical and thalamic input, several differences emerged between the datasets. Relative to Smith et al., we observed a smaller contribution from basal ganglia structures, a different distribution of cortical inputs, and prominent labeling of thalamic regions such as VM that were not emphasized in the earlier study. These differences may partly reflect methodological distinctions between first-generation and CVS-N2c rabies tracing approaches, which differ in transsynaptic efficiency and input sampling. However, they may also arise from differences in the targeted neuronal populations. Whereas Smith et al. examined patch neurons labeled in the Sepw1_NP67 line, the present study selectively targeted Kremen1^+^ patch SPNs, a molecularly defined subset of patch neurons that may exhibit specialized connectivity. This possibility is further illustrated by the minimal BNST input observed here. Although Smith et al. identified BNST projections to patch/exo-patch neurons, these inputs constituted a relatively small fraction of total afferents and may preferentially target patch subpopulations not captured by the Kremen1-Cre line. Therefore, differences in basal ganglia, limbic cortical, and thalamic input proportions may reflect specialized connectivity of Kremen1^+^ SPNs rather than the overall patch compartment. In this context, the stronger sensorimotor cortical bias and prominent VM input observed here may represent distinctive features of Kremen1^+^ SPNs. Thus, our findings complement and extend Smith et al. by refining the organization of patch-related inputs at the level of a molecularly defined SPN subtype.

Several limitations should be considered. Rabies tracing identifies anatomical connectivity but does not directly measure synaptic strength or functional influence. In addition, because input distributions were normalized to total labeled inputs per brain, our results reflect relative connectivity patterns rather than absolute input strength. Furthermore, Kremen1 marks only a subset of patch SPNs, and additional heterogeneity likely exists within this population. Although we sampled *Kremen1*^+^ SPNs across three rostrocaudal levels of the dorsal striatum, the study was designed primarily to define common whole-brain input features of this molecularly defined SPN population rather than to resolve fine-scale subregion-specific connectivity. Our exploratory comparison of major cortical input regions across rostral, medial, and caudal injection sites did not reveal significant site-dependent differences, supporting pooled analysis for the present study. However, because the number of animals per injection location was limited, subtle anatomical differences among *Kremen1*^+^ SPNs in distinct striatal subregions may not have been detected. Future studies combining circuit mapping with *in vivo* functional approaches and larger cohorts per injection coordinate will be required to determine how specific inputs shape neuronal activity and behavior and to resolve subregion-specific input differences with higher statistical power.

Because helper-virus expression is Cre dependent, cortical FLAG-positive neurons, if present, could theoretically serve as starter cells if EnvA-pseudotyped RVDG rabies virus leaked along the injection track. However, the cortical FLAG signal observed near the needle track lacked clear neuronal morphology, and we did not detect cortical cells co-labeled with FLAG and rabies reporter fluorescence. Thus, although rare cortical starter cells cannot be completely excluded, their contribution is likely minimal and unlikely to account for the broad, anatomically organized whole-brain input patterns observed in this study.

In summary, our results define the input architecture of *Kremen1*^+^ patch SPNs and support a model in which these neurons integrate widespread cortical and subcortical signals to regulate locomotor vigor and motivational state. This work advances our understanding of striatal compartmental function and highlights the importance of molecularly defined neuronal subtypes in shaping basal ganglia circuit organization.

## Significance statement

The striatum contains molecularly and functionally diverse populations of spiny projection neurons (SPNs), yet how specific subtypes are embedded within brain-wide circuits requires further study. *Kremen1*-positive (*Kremen1*^+^) SPNs are enriched in patch (striosome) compartments and have been implicated in regulating dopaminergic signaling and motor behavior. By combining genetic targeting with monosynaptic rabies tracing, this study provides the first comprehensive whole-brain map of inputs to *Kremen1*^+^ SPNs. The results reveal that these neurons preferentially integrate signals from sensorimotor cortical areas, thalamic nuclei, and neuromodulatory midbrain regions. Together with prior evidence that *Kremen1*^+^ SPNs regulate dopaminergic neurons and locomotor behavior, this connectivity profile suggests that *Kremen1*^+^ SPNs represent a molecularly defined patch-enriched SPN subtype through which cortical and thalamic signals may influence dopaminergic basal ganglia circuits, rather than a uniquely integrative architecture absent from other SPN populations.

## Data Availability

The datasets presented in this study can be found in online repositories. The names of the repository/repositories and accession number(s) can be found below: Whole-brain image datasets from all eight mice are publicly available through the Biolucida platform (https://gerfenc.biolucida.net/images/?page=images&selectionType=collection&selectionId=147).
