# Supplementary material for: Whole-brain input organization of Kremen1-positive striatal spiny projection neurons revealed by monosynaptic rabies tracing
Source: Front Neural Circuits. 2026 Jul 29;20:1895042. doi: 10.3389/fncir.2026.1895042 (PMC13462475; doi:10.3389/fncir.2026.1895042)
Supplement: Supplementary file 1 [file Data_Sheet_1.pdf]

Title: **Whole-Brain Input Organization of *Kremen1*-Positive Striatal Spiny Projection Neurons Revealed by Monosynaptic Rabies Tracing**

Authors: Victor M. Martinez Smith[1,#], Jie Dong[1,2,#], Ronald Paletzki[3,#], Lisa Chang[1], Lupeng Wang[1], Charles R. Gerfen[3], and Huaibin Cai[1]

Supplementary Figures

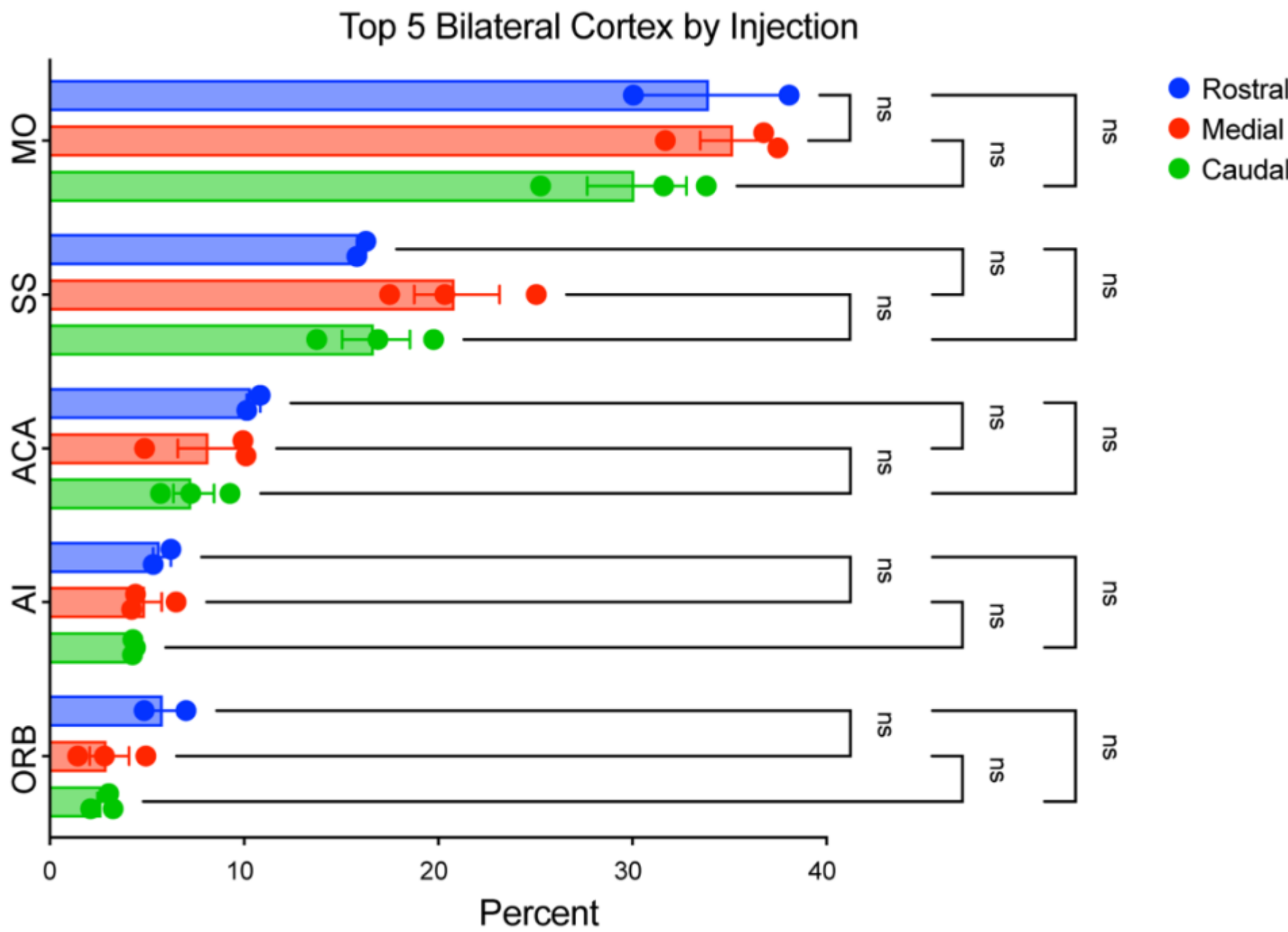

Supplementary Fig. 1. Cortical input distributions are similar across the three striatal injection sites.

Quantification of the top five bilateral cortical input regions to Kremen1⁺ SPNs for rostral, medial, and caudal striatal injection sites. Bars represent the mean percentage of total cortical input neurons from each cortical region, with individual dots indicating values from individual animals. Error bars indicate SEM. 2-way ANOVA, multiple comparisons. Across the three injection sites, the relative contributions of the major cortical input regions, including somatomotor (MO), somatosensory (SS), anterior cingulate (ACA), agranular insular (AI), and orbital cortical (ORB) areas, showed no significant differences. These results support pooling data across the three injection sites for the whole-brain input analysis presented in the main figures.

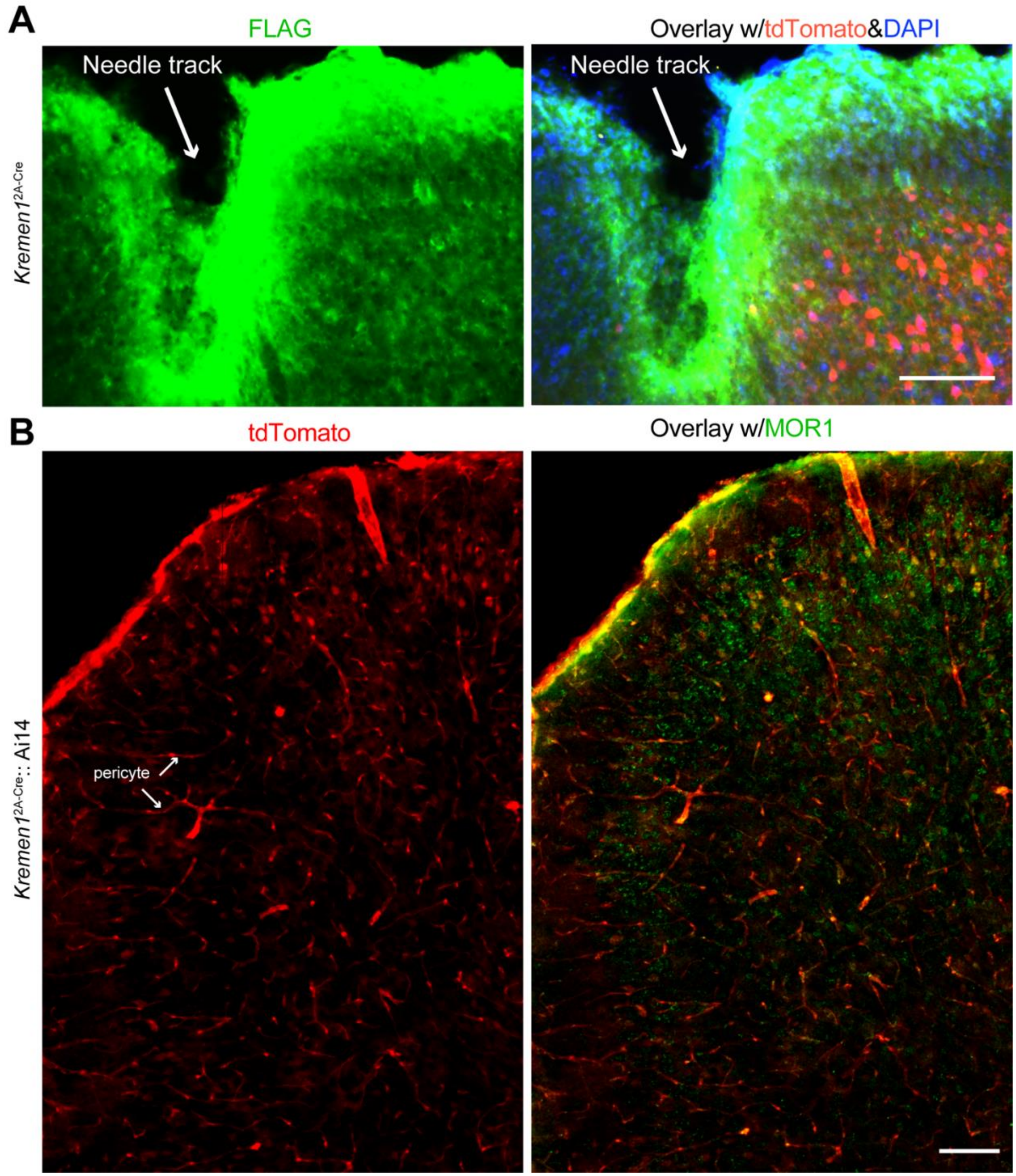

Supplementary Fig. 2. Cortical FLAG signal near the injection needle track and characterization of Kremen1-Cre expression.

(A) Higher-magnification images of the cortical region surrounding the injection needle track in Fig. 1B in a *Kremen1*$^{2A\text{-}Cre}$ mouse. FLAG immunostaining was observed along the needle track, but the signal was largely associated with damaged tissue surrounding the injection path and did not show clear neuronal morphology. Overlay with tdTomato and DAPI shows no obvious

FLAG-positive cortical starter neurons.

(B) Representative cortical images from *Kremen1*$^{2A\text{-}Cre}$; Ai14 reporter mice (Dong et al., 2025) showing sparse tdTomato labeling in the cortex. Labeled structures were mainly associated with vascular/pericyte-like morphology rather than abundant neuronal labeling. Overlay with MOR1 is shown for anatomical reference. Scale bars: 200 µm.

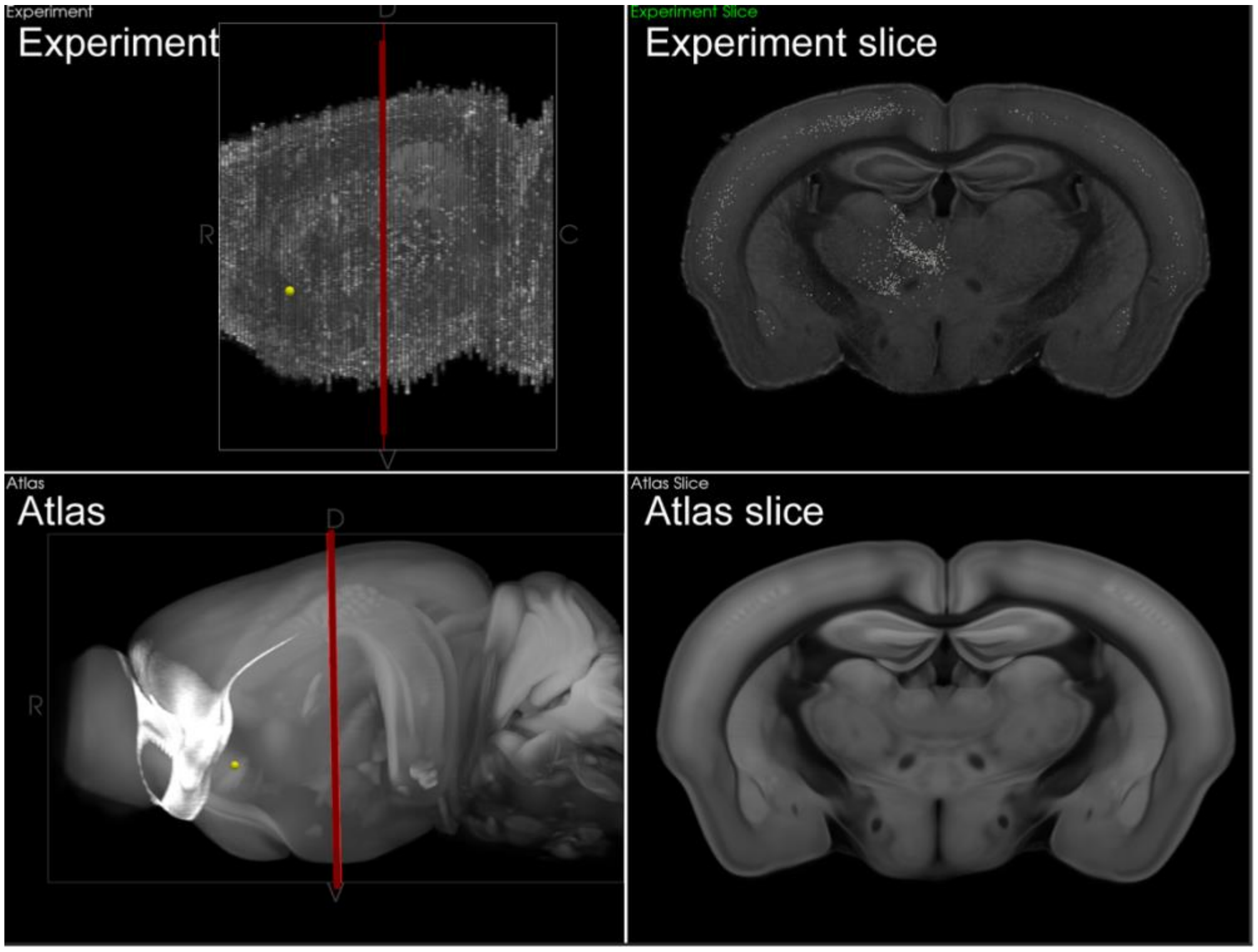

Supplementary Fig. 3. Representative NeuroInfo atlas registration of whole-brain sections. Representative registered coronal brain sections showing Allen Brain Atlas boundaries overlaid onto experimental images. Experimental sections and three-dimensional brain volumes were registered to the reference atlas using NeuroInfo. Reference sections containing prominent anatomical landmarks were first interactively aligned and then used to initialize serial registration of adjacent sections. Reference levels included a rostral section where the corpus callosum first crosses the midline, a central section where the anterior commissure appears as three distinct fiber bundles, and a caudal section corresponding to the first appearance of a rounded fasciculus retroflexus. Three-dimensional registration was then performed using both linear and nonlinear transformations. The overlay examples illustrate the spatial alignment used to assign rabies-labeled presynaptic input neurons to atlas-defined anatomical regions and subregions.
